# Cathepsin B Is Up-Regulated and Mediates Extracellular Matrix Degradation in Trabecular Meshwork Cells Following Phagocytic Challenge

**DOI:** 10.1371/journal.pone.0068668

**Published:** 2013-07-03

**Authors:** Kristine Porter, Yizhi Lin, Paloma B. Liton

**Affiliations:** Department of Ophthalmology, Duke University, Durham, North Carolina, United States of America; University of Rochester, United States of America

## Abstract

Cells in the trabecular meshwork (TM), a tissue responsible for draining aqueous humor out of the eye, are known to be highly phagocytic. Phagocytic activity in TM cells is thought to play an important role in outflow pathway physiology. However, the molecular mechanisms triggered by phagocytosis in TM cells are unknown. Here we investigated the effects of chronic phagocytic stress on lysosomal function using different phagocytic ligands (E. coli, carboxylated beads, collagen I-coated beads, and pigment). Lysotracker red co-localization and electron micrographs showed the maturation of E. coli- and collagen I-coated beads-containing phagosomes into phagolysosomes. Maturation of phagosomes into phagolysosomes was not observed with carboxylated beads or pigment particles. In addition, phagocytosis of E. coli and collagen I-coated beads led to increased lysosomal mass, and the specific up-regulation and activity of cathepsin B (CTSB). Higher levels of membrane-bound and secreted CTSB were also detected. Moreover, in vivo zymography showed the intralysosomal degradation of ECM components associated with active CTSB, as well as an overall increased gelatinolytic activity in phagocytically challenged TM cells. This increased gelatinolytic activity with phagocytosis was partially blocked with an intracellular CTSB inhibitor. Altogether, these results suggest a potential role of phagocytosis in outflow pathway tissue homeostasis through the up-regulation and/or proteolytic activation of extracellular matrix remodeling genes.

## Introduction

The trabecular meshwork (TM) is a tiny tissue located in the anterior segment of the eye, between the cornea and the sclera, which is involved in maintaining proper levels of intraocular pressure (IOP). Failure of the TM tissue commonly leads to elevated IOP, the best known causative factor for developing glaucoma, a potentially blinding disease characterized by irreversible damage to the optic nerve [1,2].

The TM is composed of sheets of connective tissue beams lined by TM endothelial cells. The beams attach to each other in several layers forming a porous filter-like structure [3,4]. Trabecular meshwork cells lining the beams are known to be highly phagocytic, capable of avidly phagocyte particulate material and debris in vitro and in vivo [5–11]. It is thought that this phagocytic activity helps keep the drainage channels free of obstructive material or debris, which otherwise might block the flow of aqueous humor and lead to elevated IOP [7]. Abnormalities in phagocytosis have been postulated to contribute to the development of certain types of glaucoma [12–14].

Several studies have shown the detachment of TM cells from the trabecular beams following phagocytosis in vivo and in vitro [5,6,8,15,16], as well as short-term loss in cell-matrix cohesiveness in cell culture conditions [17,18]. The molecular mechanisms encompassing those events still need to be fully characterized. Our laboratory very recently reported the transcriptional up-regulation of the metzincins, matrix metalloproteinase 1 (MMP1) and 3 (MMP3), as well as increased collagenase activity in cultured TM cells following phagocytosis to E. coli and autologous pigment particles, which might explain the loss in cell-matrix cohesiveness upon phagocytosis [19]. However, although it has been historically believed that MMPs are the major proteases involved in extracellular matrix (ECM) degradation, novel research data seem to contradict this central dogma and suggest that, while MMPs might play a critical regulatory role in ECM metabolism, other proteases or the coordinated action of several types of proteases are responsible for the bulk matrix degradation [20].

According to their catalytic mechanisms, proteases are classified into six different groups: serine proteases, threonine proteases, cysteine proteases, aspartate proteases, glutamic acid proteases, and metalloproteinases [21]. Different types of proteases have different action mechanisms and biological processes [22]. Of special interest are lysosomal cysteine cathepsins given the close relationship between the phagocytic and the lysosomal pathways. Phagocytosed material is engulfed and internalized within a membrane-bound organelle, the phagosome, which subsequently fuses with the lysosome, forming the phagolysosome, whereby the ingested material is degraded by lysosomal hydrolytic enzymes, also known as cathepsins [23–26]. Although cathepsins have optimal activity at lysosomal acidic and reducing environment, they have been shown to significantly contribute to the degradation of the ECM under physiological and pathological conditions [20,27,28].

In this study, we wanted to investigate the effects of chronic phagocytic stress on lysosomal function, and whether altered lysosomal function elicited by phagocytosis could contribute to the loss in cell-matrix cohesiveness and increased collagenolytic activity observed in TM cells upon phagocytosis. Here we report for the first time the specific up-regulation of cathepsin B (CTSB) and the CTSB-mediated degradation of the ECM substrate gelatin upon phagocytosis of E.coli and collagen I-coated beads in TM cells. Our data support a novel role of phagocytic function in TM tissue homeostasis. 

## Experimental Procedures

**Reagents**. pHrodo^TM^ E. coli bioparticles®, FITC-labeled E. coli and FluoSpheres® collagen I-labeled microspheres (1.0 µm, yellow-green fluorescent) were obtained from Invitrogen (Carlsbad, CA); Fluoresbrite® Blue (BB) carboxylate microspheres (1 μM diameter) were obtained from Polyscience Inc (Warrington, PA); lysotracker red (LTR) was obtained from Invitrogen. Ca074Me was obtained from Enzo Life Sciences (Farmingdale, NY). Casein, plasminogen, EDTA, E64, and PMSF were obtained from Sigma-Aldrich (St. Louis, MO).

### Isolation of Porcine Trabecular Meshwork Cells

Primary cultures of porcine TM cells were prepared and maintained as previously described [29]. All the experiments were performed using three different cell lines at passage four.

### Pigment Isolation

Isolation of porcine pigment particles was performed as previously described [19]. Briefly, the anterior segment of a porcine eye was separated from the posterior pole. The iris and ciliary body were dissected and gently mixed in 40 ml sterile water and centrifuged at 120 x g for five minutes to eliminate cell debris. The supernatant was further centrifuged at 800 x g for 15 minutes. Pigment pellets were resuspended in PBS and stored at -80 ^o^C. The concentration of pigment particles was calculated with the use of a hemocytometer.

### Aqueous humor sample collection

Porcine aqueous humor was collected through trans-corneal injection in pig cadaver eyes obtained under approval from a local slaughterhouse (City Packing CO, Burlington, NC) less than five hours post-mortem, clarified by centrifugation at 1500 g x 5 min at 4 °C to remove cell debris, and stored at -80 °C until use.

### Chronic phagocytic challenge to TM cells

Confluent cultures of porcine TM cells were phagocytically challenged to 1 x 10^6^ particles/mL of either pHrodo^TM^ E. coli bioparticles®, FITC-labeled E. coli, Fluoresbrite® Blue carboxylate microspheres (1 μM diameter) or autologous iris pigment, added twice per week to fresh completed culture media for up to ten days. Phagocytic activity was monitored under the brightfield and fluorescence microscopes. Experiments were performed at the indicated times post-initial challenge.

### Quantification of Phagocytic Activity

Phagocytic activity was quantified by flow cytometry using E. coli bioparticles labeled with the pH-sensitive dye pHrodo (pHrodo^TM^ E. coli bioparticles®, Invitrogen, Carlsbad, CA), which is non-fluorescent outside the cell, but becomes fluorescence in the acidic environment of the phagosome. For this, confluent cultures of porcine TM cells were phagocytically challenged with pHrodo-labeled E.coli (1 x 10^6^ particles/mL) as detailed above. At days 2, 5 and 10 post-initial phagocytic challenge, cells were washed in PBS, trypsinized, and resuspended in single cells solution. The orange/red fluorescence emitted by 10,000 cells in the FL-2 channel was recorded and analyzed (CellQuest software; BD Biosciences).

### Quantification of Lysosomal Mass

The lysosomal cellular content was evaluated using the lysosomotropic dyes, Lysotracker Red [LTR] (Invitrogen, Carlsbad, CA) as follows. Confluent cultures of porcine TM cells were phagocytically challenged to FITC-labeled E. coli, Fluoresbrite® Blue carboxylate microspheres (1 μM diameter) or autologous iris pigment as detailed above. At days 2, 5, and 10 post-initial phagocytic challenge, cells were incubated with 100 nM LTR in fresh media for one hour at 37 ^o^C, 5% CO_2_. Specific lysosomal labeling was confirmed by fluorescence microscopy. LTR fluorescence was quantified by flow cytometry in the red spectrum (FL-3 channel).

### Phagosome Maturation

Maturation of phagosomes into phagolysosomes was monitored using LTR. For this, PTM cells exposed to phagocytic challenge to either FITC-labeled E. coli or Fluoresbrite® Blue (BB) carboxylate microspheres for three days were incubated with 100 nM LTR in fresh media for one hour at 37 ^o^C, 5% CO_2_. Cells were then washed in PBS and incubated for two minutes with Trypan Blue (0.5 mg/mL) to quenched fluorescence from non-engulfed and surface-bound particles. After washing, cells were fixed in 4% paraformaldehyde for 10 min at room temperature. Co-localization of phagocytic ligands within the lysosomal compartment was evaluated by confocal microscopy (Nikon Eclipse 90i).

### Electron microscopy

Cells were washed twice in PBS and fixed in 2.5% glutaraldehyde in 0.1M cacodylate buffer (pH 7.2). Fixed cells were then detached by gentle scraping, pelleted, post-fixed in 1% osmium tetroxide in 0.1M cacodylate buffer, and processed for transmission electron microscopy in the Morphology Facility at Duke Eye center. Thin sections (65 nm) were examined in a JEM-1200EX electron microscopy.

### Electrophoresis and Western-blot

To obtain whole-cell lysates, cells were washed in PBS and lysed in 20 mM Hepes, 2mM EGTA, 5mM EDTA, 0.5% NP-40 containing Halt Protease Inhibitor Cocktail and Halt Phosphatase Inhibitor Cocktail (Pierce, Rockford, IL). Protein concentration was determined using Micro BCA Protein Assay Kit (Pierce, Rockford, IL). Cell surface proteins were purified by selective biotinylation using the Cell Surface Protein Isolation Kit (Pierce, Rockford, IL), following the manufacturer’s instructions. Protein samples (5-20 μg whole cell lysates, 20 μg cell surface fraction, 25 μL conditioned media) were separated by 10% SDS-PAGE and transferred to PVDF membrane (Bio-Rad, Hercules, CA). Membranes were blocked with 5% nonfat dry milk and incubated overnight with primary antibody diluted in blocking solution containing 0.05% Tween 20. Bands were detected by incubation with a secondary antibody conjugated to horseradish peroxidase and chemiluminescence substrate (ECL Plus, GE Healthcare, Pittsburgh, PA). The source of the primary antibodies are the following: anti-CTSB (ab58802-100) and anti-LAMP1 (ab24170) from Abcam (Cambridge, MA), anti-cathepsin D (SC-6494), anti-cathepsins (SC-6499), chi3L1 (SC-30465) or anti-tubulin (SC-9935) from Santa Cruz Biotechnology (Sta. Cruz, CA).

### RNA Isolation and qPCR

Total RNA from TM primary cultures was isolated using RNeasy kit (Qiagen, Valencia, CA), following the manufacturer’s protocol, and then treated with DNase I. RNA yields were determined using the RiboGreen fluorescent dye (Molecular Probes, Eugene, OR). First-strand cDNA was synthesized from total RNA (1 μg) by reverse transcription using oligo(dT) primer and Superscript II reverse transcriptase (Invitrogen, Carlsbad, CA). Real-time PCRs were performed as previously described. The fluorescence threshold value (Ct) was calculated using the iCycle iQ system software. The average Ct value of the following housekeeping genes (β-Actin, GAPDH, and HPRT1) served as internal standard of mRNA expression. The fold change was calculated using the formula 2^-ΔΔCt^, where Δ_Ct_=Ct_gene_-Ct_average housekeeping_, and ΔΔ_Ct_=Δ_CtExp_-Δ_CtCon_. The sequences of the primers used for the amplifications are shown in Table 1.

**Table 1:** Primer Sequences Used for qPCR Analysis.

**Gene Symbol**	**Forward**	**Reverse**
CTSB	GCAACTCCTGGAACACAGAC	CCACGATCTCTGACTCGATG
CTSD	CAGAAGCTGGTGGACAAGAA	GCGTGACGTTGTGATAGTCC
CTSK	ACGTATGAACTGGCCATGAA	ATTACTGCGGGAATGAGAGG
CTSL	GGCAAGCTTGTTTCACTGAG	CCTCCATTGTCCTTCACGTA
β-Actin	TCCCTGGAGAAGAGCTACGA	AGGAAGGAAGGCTGGAAGAG
GAPDH	TGTCCCCACCCCCAACGTGT	CCCTCGGACGCCTGCTTCAC
HRPT1	ACACTGGCAAAACAATGCAA	ACACTTCGAGGGGTCCTTTT

### Quantification of Cathepsin Activities

Cells grown in a 24-well plate were washed in PBS and lysed for 30 minutes at 4°C with shaking in 100 μL of 50 mM sodium acetate (pH 5.5), 0.1 M NaCl, 1 mM EDTA, and 0.2% Triton X-100. Lysates were clarified by centrifugation and immediately used for determination of proteolytic activity. For this, 1 μL of cell lysates was incubated at 37°C for 30 minutes in lysis buffer (100 μL) in the presence of the appropriate fluorogenic substrate. The following cathepsin substrates were used in this study: z-FR-AMC (20 μM; Santa Cruz Biotechnology), z-RR-AMC (20 μM), z-VVR-AMC (20 μM), z-GPR-AMC (20 μM), and Cathepsin D & E substrate (10 μM); all from Enzo Life Sciences (Farmingdale, NY). The AMC released as a result of proteolytic activity was quantified with a microtiter plate reader (exc: 380 nm; em: 440 nm), and normalized by total protein content. The MCA released as a result of cathepsin D & E proteolytic activity was read at 340 nm (exc) and 420 nm (em).

### Construction of CTSB-GFP Plasmid and Transfection

Human CTSB cDNA non-containing the STOP codon was amplified from MGC Clone BC095408.1, obtained from the Mammalian Gene Collection (NIH), by high-fidelity PCR (Advantage HF 2 PCR kit, Clontech, Mountain View, CA) using the following primers: forward (GGCTCGAGACATGTGGCAGCTCTGGGCCT) and reverse (GCGTCGACGATCTTTTCCCAGTACTGATCG) containing the restrictions enzyme sites *Xho*I and *SalI*, respectively. The PCR fragment product was cloned into pCR 2.1-TOPO (Invitrogen, Carlsbad, CA). The absence of mutations confirmed by full-length sequencing. CTSB was then digested with XhoI and SalI and cloned into pEGFP-N3 (Clontech, Mountain View, CA) to generate pCTSB-GFP. Primary cultures of porcine TM cells were transiently transfected with pCTSB-GFP (2 μg) by electroporation with the Nucleofector System (T23 program, Amaxa Inc., Gaithersburg, MD) using the Basic Nucleofector® Kit for Primary Endothelial Cells, according to the manufacturer’s instructions.

### Cell Viability

Cell viability was quantified with a cytotoxicity assay (CytoTox 96 Non-Radioactive Cytotoxicity Assay; Promega) that measures the lactate dehydrogenase (LDH) release on cell lysis in accordance with the manufacturer’s instructions.

### Substrate Gel Zymography

Gelatin, casein, and plasminogen/casein zymography were performed as follows. Serum-free cell culture supernatant samples (25 μl) were mixed with equal volumes of 2X zymography sample buffer (125 mM Tris-HCl, pH 6.8, 50% glycerol, 8% SDS, 0.02% bromophenol blue), loaded onto SDS-PAGE gels containing gelatin, casein, or casein/plasminogen under nonreducing conditions, and electrophoresed with 2.5 mM Tris-HCl, 19.2 mM glycine, 0.01% SDS, pH 8.3, at 100 V. After electrophoresis, gels were washed with 1x renaturing buffer and 1x development buffer for 30 minutes each, and incubated overnight in zymogram development buffer (Bio-Rad). Gels were then stained with Coomassie blue R-250 followed by destaining with 55% methanol and 7% acetic acid. Areas of MMP activity appeared as clear bands. Pre-formulated zymography buffers and pre-cast gelatin and casein gels were purchased from Bio-Rad (Hercules, CA). Plasminogen/casein-containing gels were prepared in the laboratory by adding plasminogen (13 μg/mL) and casein (1 μg/mL) to 12% acrylamide gels. To investigate the nature of the lytic bands, EDTA (20 mM), E64 (20 mM) or PMSF (1 mM) was added to the development buffer.

### Quantification of Gelatinase Activity

Gelatinase activity was monitored using DQ gelatin fluorescein conjugate (Invitrogen, Carlsbad, CA) as follows. Confluent cultures of porcine TM cells grown in 96-well plate were phagocytically challenged to E. coli in the presence of vehicle or DQ-substrate (10 μg/mL). Fluorescence peptides released by the enzymatic cleavage of the substrate were measured in a microplate reader at the indicated times (Em: 495 nm; Exc: 515 nm). All values were corrected for background fluorescence. To evaluate the role of CTSB, Ca074Me (40 μM), a selective CTSB pro-inhibitor the specific intracellular CSTB inhibitor, was added to the culture media together with the vehicle or DQ-substrate throughout the duration of the experiment.

### In Situ Zymography

Live cell proteolysis was assayed as described below. Cells were grown onto Lab Tek II chambers coated with 20 μg/mL of either DQ-gelatin, DQ-Collagen I or DQ-Collagen IV (Invitrogen, Carlsbad, CA). Fluorescence peptides released by the enzymatic cleavage of the substrate were observed in live cells under confocal microscopy (Nikon Eclipse 90i). Fluorescence intensities were quantitated from five confocal fluorescence images using ImageJ.

### In Vivo Visualization of CTSB Activity

Cathepsin B activity was visualized in whole living cells using the Magic Red^TM^ Cathepsin B Assay Kit (Immunochemistry Technologies, Bloomington, MN), which utilizes the fluorophore cresyl violet linked to two CTSB target sequence peptides (Arginine-Arginine, R–R). Following CTSB-associated enzymatic hydrolysis, the two R–R peptide sequences are cleaved from the Magic Red molecule, converting it to the red fluorescent form. Briefly, cells grown in Lab-Tek II chambers were treated as indicated in each particular experiment and then incubated for one hour with 10 μL of reconstituted MR-

(RR)_2_. Cathepsin B activity was visualized by in vivo confocal microscopy (Nikon Eclipse 90i). Fluorescence intensities were quantitated from five confocal fluorescence images using ImageJ.

### Statistical Analysis

All experimental procedures were repeated at least three times in independent experiments using three different cell lines. The percentage of increase of the experimental conditions compared to the control was calculated and averaged. Data are represented as mean ± SD. Statistical significance was calculated using Student’s t-test for two groups comparisons using the software GraphPad Prism. A probability less than 5% was considered statistically significant.

## Results

### Trabecular meshwork cells are capable of internalizing different phagocytic ligands for extended period of time without compromising cell viability

**Figure 1:**
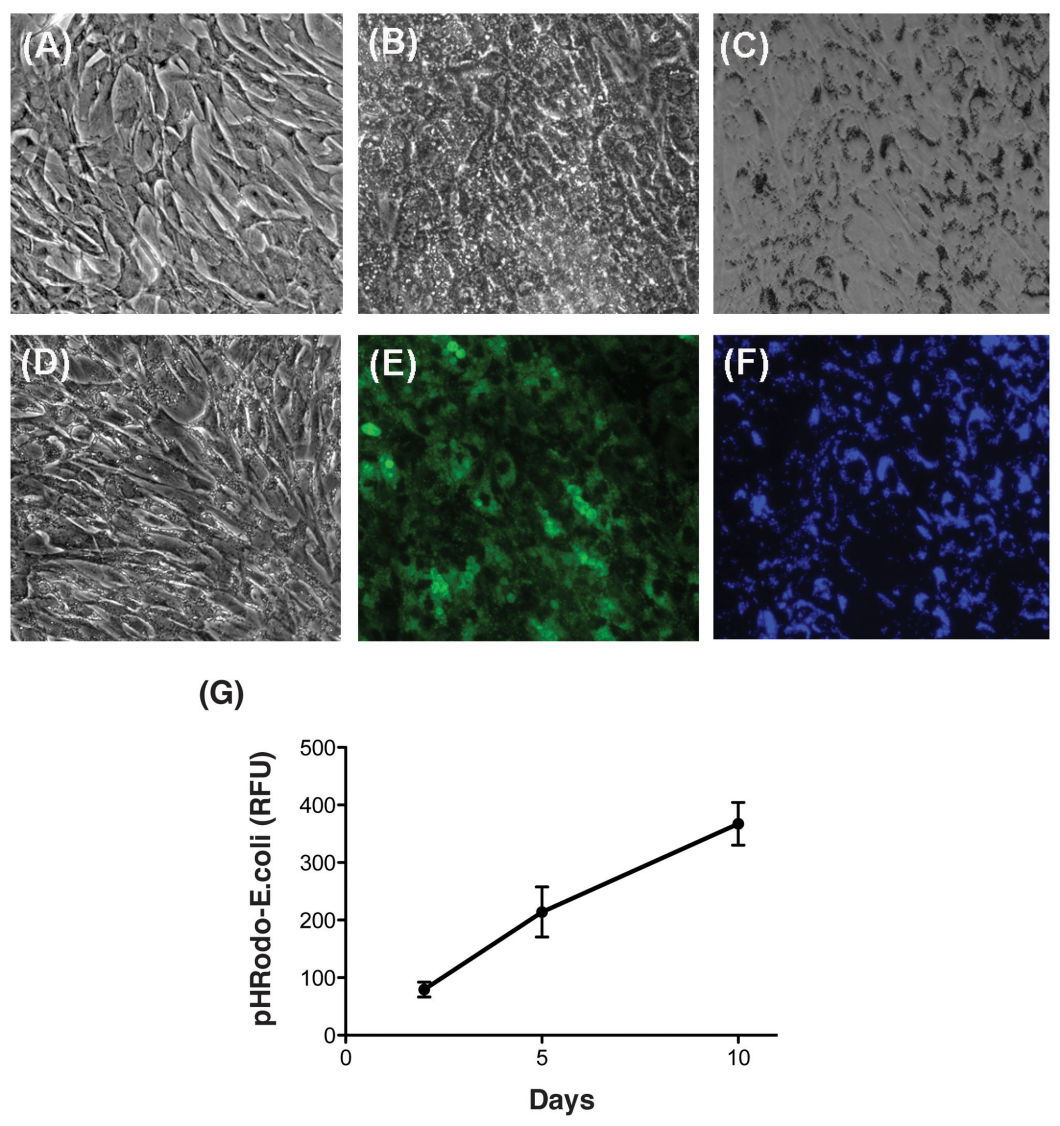
Phagocytic activity in TM cells. Representative brightfield (A–D) and fluorescence microscopy (E, F) images of control TM cells (A) or TM cells phagocytically challenged for ten days to 1 x10^6^ particles/mL of (B, E) FITC-labeled E. coli; (C, F) Fluoresbrite® Blue carboxylate microspheres, 1 μM diameter; and (D) autologous iris pigment. (G) Flow cytometry quantification of internalized pHRodo-labeled E.coli particles overtime.

To evaluate the effects of chronic phagocytic stress on lysosomal function, confluent cultures of porcine TM cells were phagocytically challenged to biotic and non-biotic phagocytic ligands (FITC-labeled E.coli, Fluoresbrite® Blue carboxylate microspheres, and autologous iris pigment) twice per week in completed culture media, and kept under normal culture conditions for up to ten days. In some instances, cells exposed to E. coli showed a temporary slight loss in adhesivity to the substrate during the first hours following addition of the ligand, which was characterized by cell rounding. However, they soon re-attached to the plate and remained as a monolayer during the duration of the experiment (Figure 1A–D). As shown in Figure 1E-F, more than 90% of the cells displayed phagocytic capacity against both, biological and inert ligands. Microscopical observation and quantification by flow cytometry indicated a linear increase in both, the number of cells engaged in phagocytosis and the amount of internalized material overtime (Figure 1G).

### Internalized *E. coli* particles, but not carboxylated beads or pigment particles, are delivered to the lysosomes and increase lysosomal mass in TM cells

**Figure 2:**
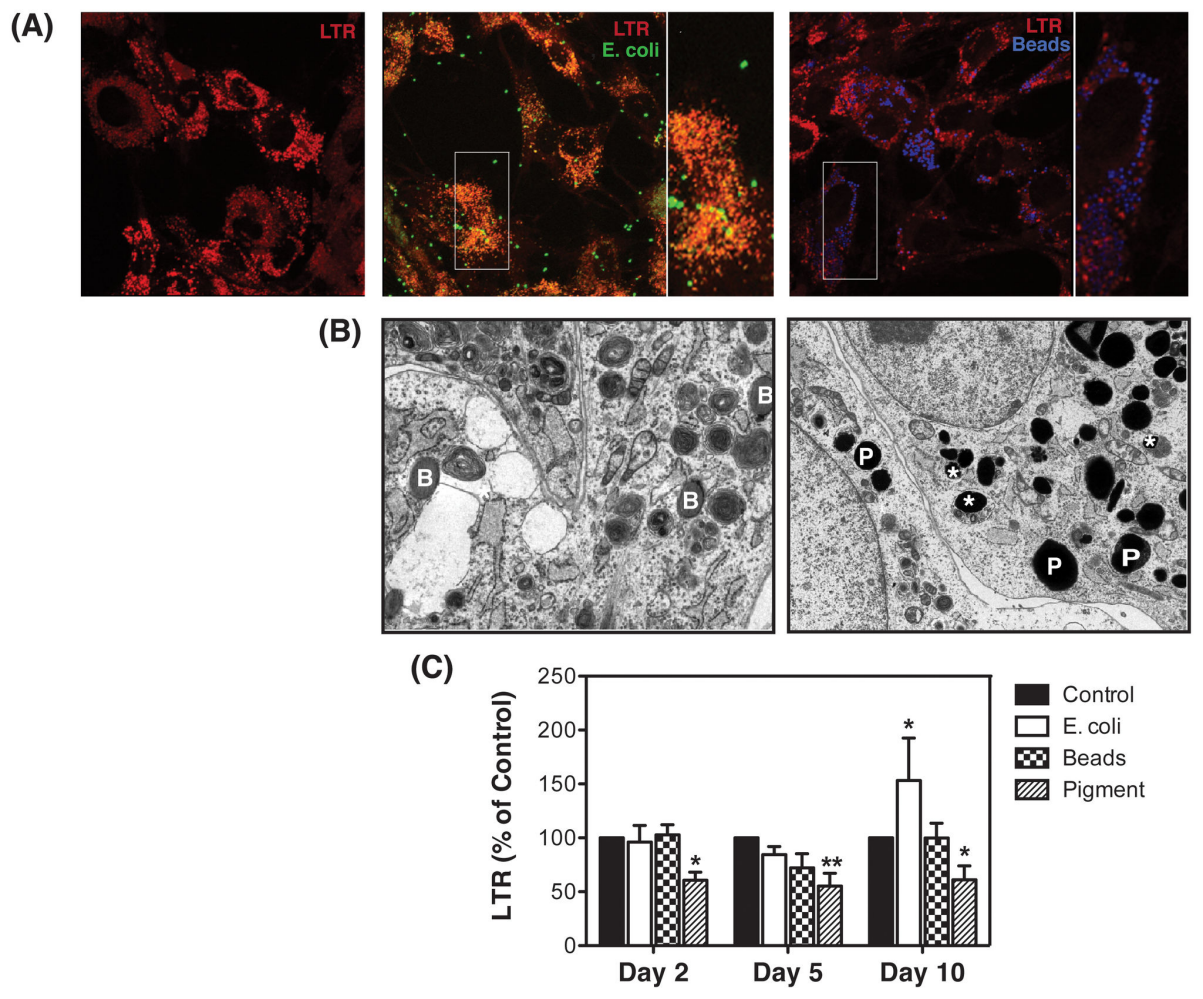
Internalized E. coli particles but not carboxylated beads are delivered to the lysosomes and increase lysosomal mass in TM cells. (A) Confluent cultures of TM cells were subjected for three days to phagocytic challenge to either FITC-labeled E.coli (left panel) or Fluoresbrite® blue carboxylate microspheres (right panel), and then incubated with LTR (100 nM) for one hour at 37 ^o^C. Co-localization of E. coli or carboxylated beads with LTR was observed under confocal microscopy. Images are representative of three independent experiments. (B) Representative electron microscopy images of TM cells phagocytically challenged for three days to either carboxylate microspheres (left panel) or pigment particles (right panel). *B* = beads in isolated phagosomes; *P* = pigment in isolated phagosomes; * = phagolysosome containing engulfed particle. (C) Lysosomal mass quantified by flow cytometry in the FL3 channel of TM cells phagocytically challenged to 1 x10^6^ particles/mL of either FITC-labeled E. coli, Fluoresbrite® blue carboxylate microspheres or autologous iris pigment. Data is represented as percentage of control. Values are means ± SD. *p<0.05; **p<0.001 (t-test, n=3).

To confirm maturation of phagosomes into phagolysosomes, we used the lysosomal marker LTR. As shown in Figure 2A, FITC-labeled E. coli bioparticles colocalized with LTR (Figure 2A, left panel, orange punctuated staining resulting from co-localization of green and red signals). In contrast, just a small number of carboxylated microspheres were found to co-localize within phagolysosomes (Figure 2A, right panel, magenta punctuated staining resulting from co-localization of blue and red signals). Electron microscopy analysis further confirmed this observation. As illustrated in Figure 2B, carboxylated beads (B, left panel) and pigment particles (P, right panel) were preferentially located within isolated phagosomes, without undergoing fusion with lysosomes. Just a small number of pigment-containing phagosomes showed maturation into autophagolysosomes, as indicated by the presence of cytosolic and membranous material (marked with asterisks, right panel).

Flow cytometry analysis showed increased lysosomal mass in cultures phagocytically challenged to E. coli at day ten compared to control cultures (153.21 ± 39.35%, p<0.05, n=3, Figure 2C). The engulfment of pigment particles, however, significantly reduced the fluorescence levels associated to LTR at all the times tested (84.44 ± 7.61% at day 2, p<0.05; 72.05 ± 13.13% at day 5, p<0.001; 55.27 ± 27% at day 10, p<0.05, n=3). No changes in lysosomal content were observed with carboxylated beads.

### Phagocytic challenge to *E. coli* but not to pigment particles or carboxylated beads induces the upregulation of CTSB expression in TM cells

**Figure 3:**
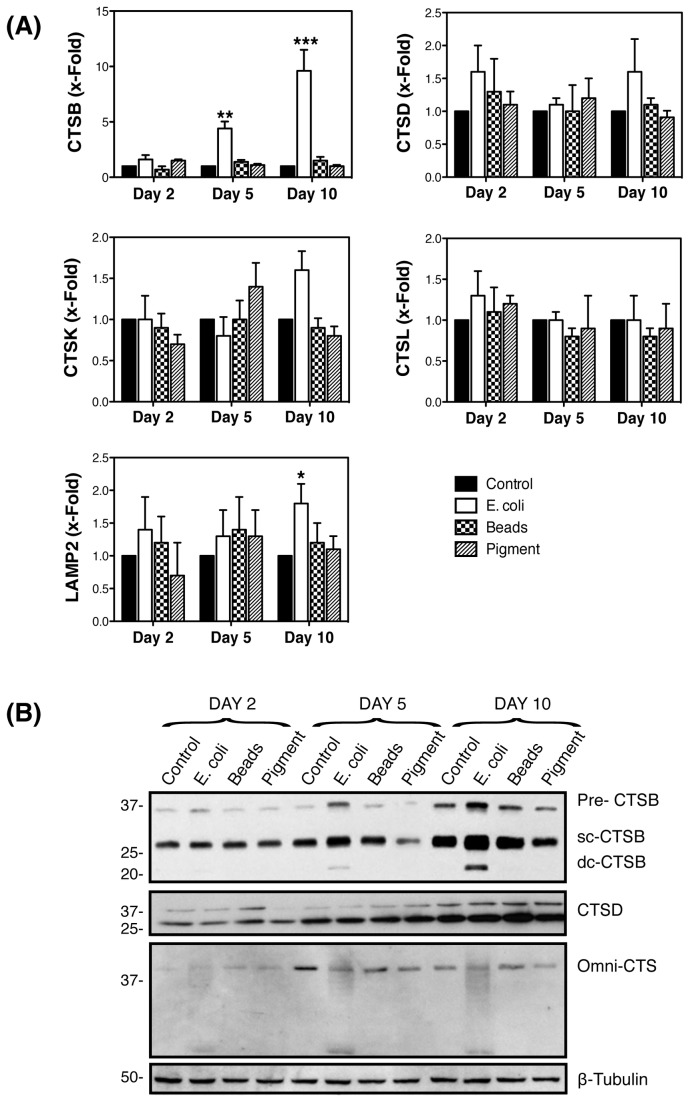
Phagocytic challenge to E. coli upregulates the expression of CTSB expression in TM cells. Confluent cultures of TM cells were phagocytically challenged to 1 x10^6^ particles/mL of either FITC-labeled E. coli, Fluoresbrite® blue carboxylate microspheres or autologous iris pigment. (A) mRNA expression levels of CTSB, CTSD, CTSK, and CTSL at 2, 5, and 10 days as quantified by qPCR using specific primers. β-Actin, GAPDH, and HPRT1 served as internal standard for normalization. Values are mean ± SD. **, p<0.01, ***, p <0.001 (t-test, n = 3). (B) Protein levels of CTSB, CTSD, and cathepsins evaluated by western-blot analysis in whole cell lysates (10 μg) using specific antibodies. β-Tubulin levels were used as loading control. Western-blots images are representative from three different experiments. scCTSB=single-chain CTSB; dcCTSB=double-chain CTSB.

We next examined whether the higher demand for lysosomal degradation under chronic phagocytic challenge might lead to an increase in lysosomal cathepsins. As shown in Figure 3A, exposure of porcine TM cells to E. coli bioparticles significantly induced a sustained increase in the mRNA levels of CTSB over time (1.6 ± 0.7 fold, 4.4 ± 1.1 fold, 9.6 ± 3.3 fold at days 2, 5, and 10, respectively, p<0.0001, n=3). Expression levels of CTSD, CTSK, CTSL were not significantly altered. Also, neither latex beads nor pigment promoted changes in the mRNA levels of any of the cathepsins tested.

Western-blot analysis revealed higher amounts of CTSB protein exclusively in the cells phagocytically challenged to E. coli (Figure 3B). This increased was observed in both, the inactive immature form (pro-CTSB), as well as in the mature active forms, single-chain CTSB (sc-CTSB) and double-chain CTSB (dc-CTSB), resulting from the proteolytic cleavage of the pro-enzyme in late endosomes and lysosomes, respectively. No significant changes in protein levels were observed with any of the phagocytic ligands using a specific antibody against CTSD or a omni-cathepsins antibody.

**Figure 4:**
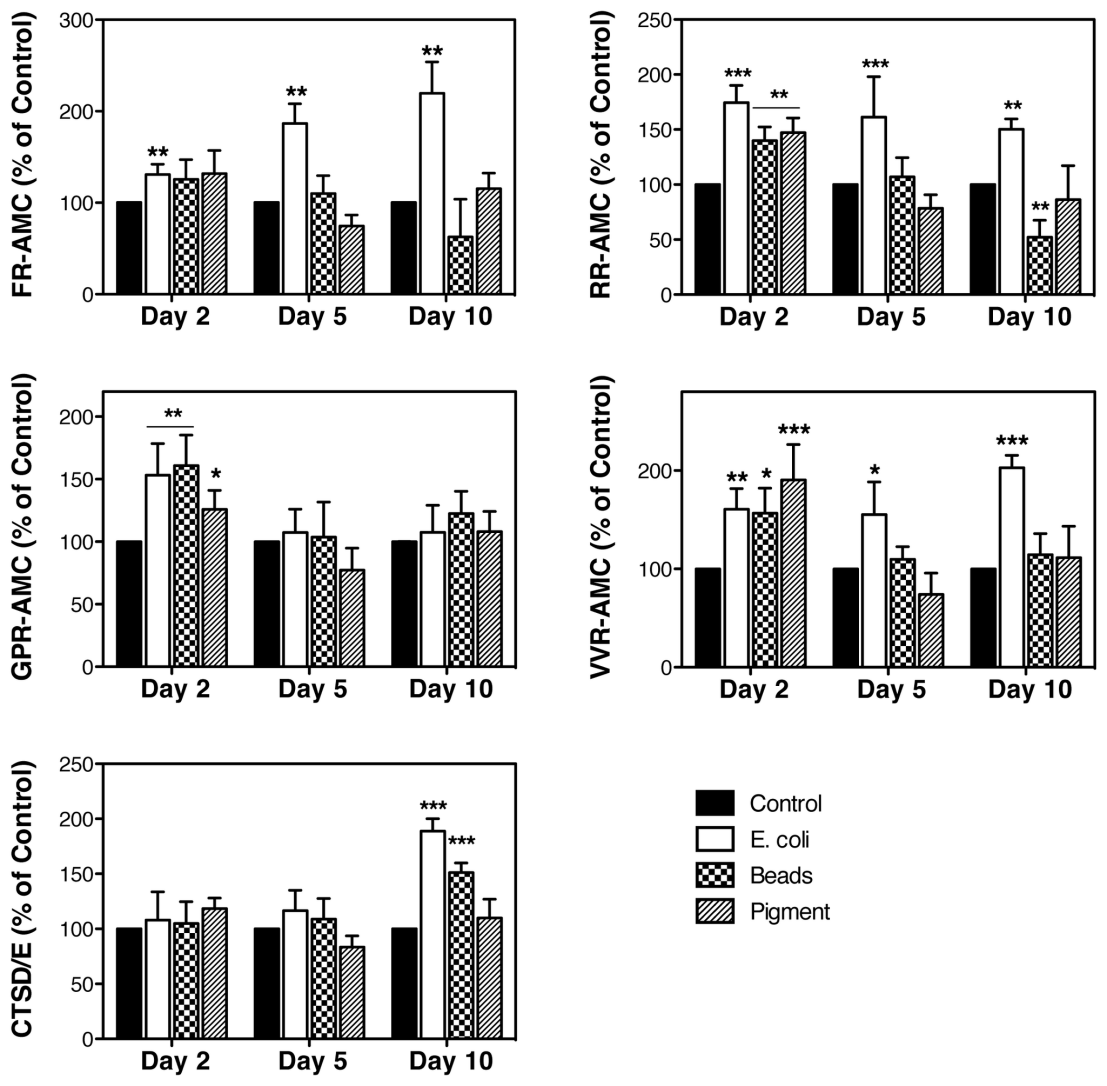
Increased cathepsin activities in phagocytically-challenged TM cells. Confluent cultures of TM cells were phagocytically challenged for 2, 5, and 10 days to 1 x10^6^ particles/mL of either FITC-labeled E. coli, carboxylate microspheres or iris pigment. Proteolytic activity in the presence of specific fluorogenic substrates. z-FR-AMC (20 μM) was used to monitor cysteine and serine proteases (kalikrein, plasmin), z-RR-AMC (20 μM) was used to monitor CTSB activity; z-VVR-AMC (20 μM) was used to monitor CTSS activity; z-GPR-AMC (20 μM) was used to monitor CTSS and CTSL activities; and CTSD/E (10 μM) to monitor aspartyl protease activity (CTSD, CTSE). Data is represented as percentage of control. Values are mean ± SD. *, p<0.05; **p<0.01, ***p<0.001 (t-test, n=3).

The activities of several serine (FR-AMC), cysteine (RR-AMC, GPR-AMC, VVR-AMC) and aspartyl proteases (CTSD/E) were quantified using the fluorogenic substrates indicated in parentheses. As shown in Figure 4, an overall higher cysteine cathepsin activity was observed with all the ligands at day 2, but returned to control levels afterwards. Sustained elevated CTSB and CTSS activities throughout the experiment were, however, detected upon phagocytosis of E. coli (RR-AMC, VVR-AMC). Phagocytic challenge to E. coli also induced a progressive significant increased over time in serine proteases activity, which include cathepsins, kalikrein, and plasmin (FR-AMC). In contrast, the activity of the aspartyl proteases CTSD and CTSE was unaltered with phagocytosis during at day 2 and 5, but significantly increased at day 10.

### Phagocytosis of collagen-coated beads also leads to upregulated CTSB expression and activity in TM cells

Our results indicate that phagocytic challenge of porcine TM cells to E. coli up-regulates the expression of CTSB. This is not observed upon phagocytosis of inert particles, such as pigment or latex beads. Production of CTSB has been reported to be induced by lipopolysaccharide, the major component of the outer membrane of Gram-negative bacteria [30]. We next wanted to investigate whether CTSB was also up-regulated upon phagocytosis of other biodegradable phagocytic ligand which do not contain LPS. For these experiments, we chose collagen I-coated microspheres, a well-established model to study collagen phagocytosis, more relevant to outflow pathway physiology.

**Figure 5:**
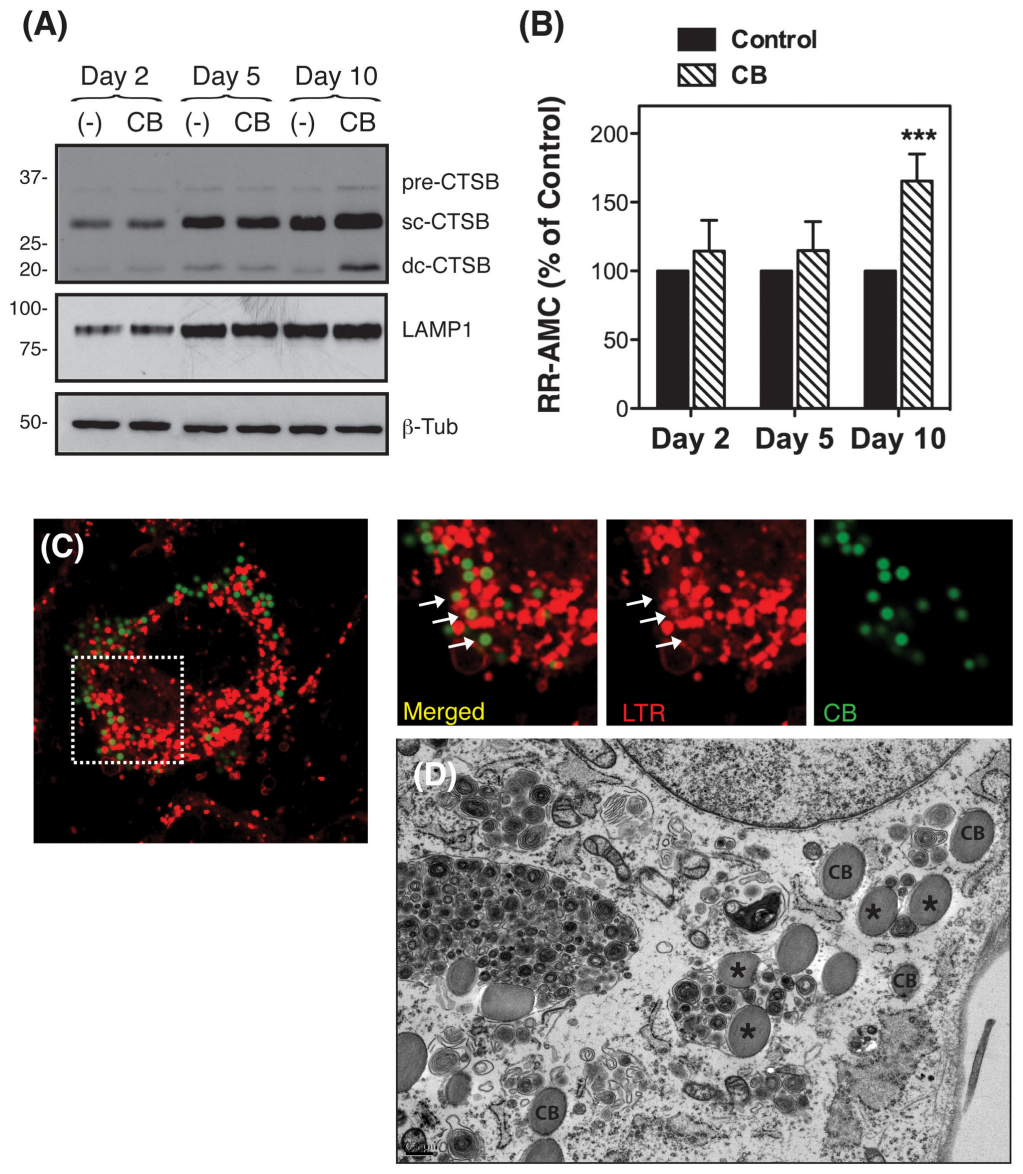
Phagocytosis of collagen I-coated beads also upregulates CTSB expression and activity in TM cells. Confluent cultures of TM cells were phagocytically challenged for 2, 5, and 10 days to FluoSpheres® collagen I-labeled microspheres (1.0 µm, 1 x10^6^ particles/mL). (A) Protein levels of CTSB and LAMP1 as evaluated by western-blot analysis in whole cell lysates (10 μg) using a specific antibody. β-Tubulin levels were used as loading control. Western-blots images are representative from three different experiments. (B) Proteolytic CTSB activity normalized by total protein content using the fluorogenic substrates z-RR-AMC (20 μM). Data is represented as percentage of control. Values are mean ± SD. ***p<0.001 (t-test, n=3). (C) Confluent cultures of TM cells were subjected for three days to phagocytic challenge to FluoSpheres® collagen I-labeled microspheres and then incubated with LTR (100 nM) for one hour at 37 ^o^C. Co-localization of collagen I-coated beads (green color) with LTR (red color) was observed under confocal microscopy. Images are representative of three independent experiments. (D) Representative electron microscopy images of TM cells phagocytically challenged for three days to FluoSpheres® collagen I-labeled microspheres. CB: beads contained in isolated phagosomes; asterisks (*): beads contained in mature autophagolysosomes.

As seen in Figure 5, phagocytosis of collagen I-coated beads significantly increased at day ten the protein levels of CTSB (Figure 5A), in particular the active double chain form. Engulfment of collagen-coated beads also led to elevated CTSB activity compared to controls (165.45 ± 19.66%, p<0.0001, n=3) (Figure 5B). In contrast to that observed for carboxylated beads, LTR staining indicated the presence of collagen-coated beads both in isolated phagosomes, non-stained with LTR (Figure 5C), as well as in LTR-containing phagolysosomes (Figure 5C, arrows). Electron microscopy analysis confirmed the presence of collagen-coated beads in both, phagosomes (Figure 5D, CB) and autophagolysosomes (Figure 5D, asterisks).

### Phagocytosis Increases Cell Surface Expression and Secretion of CTSB in TM Cells

**Figure 6:**
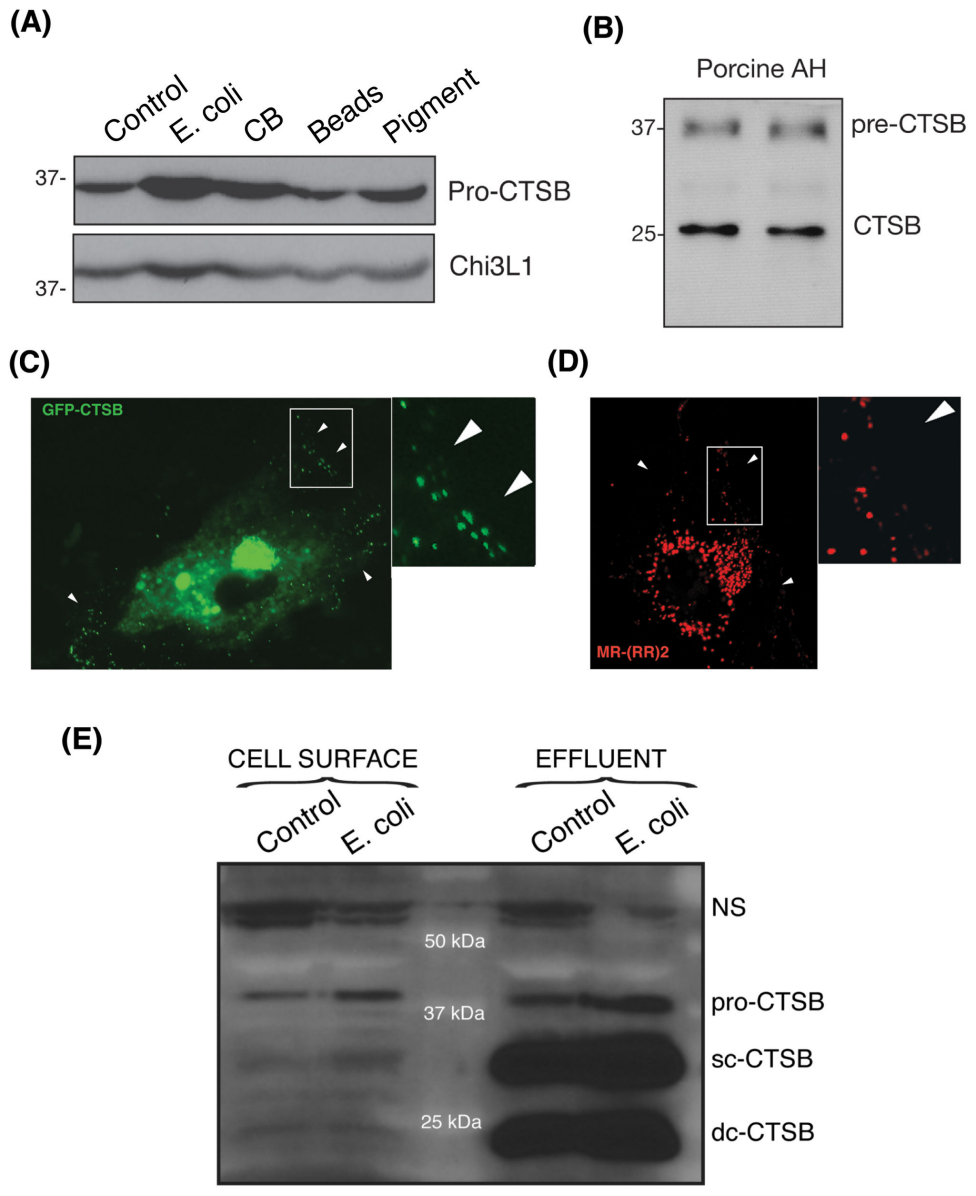
Phagocytosis Increases Cell Surface Expression and Secretion of CTSB in TM Cells. (A) Protein levels of CTSB evaluated by western-blot analysis in conditioned media (15 µL) from TM cells phagocytically challenged for ten days to either FITC-labeled E. coli, collagen I-coated beads, carboxylate microspheres, or iris pigment. The levels of the non-related protein, Chi3L1, were used as loading control. (B) Protein levels of CTSB analyzed by western blot in clarified aqueous humor samples (15 µL) collected from porcine cadaver eyes. (C) Constitutive Porcine TM cells were transfected with 2 µg of the plasmid GFP-CTSB. Two days after transfection cells were fixed, and GFP fluorescence analyzed under confocal microscopy. White arrows indicate the presence of GFP signal at the periphery of the cell. (D) Cathepsin B activity as visualized by in vivo confocal microscopy in porcine TM cells incubated for one hour with 10 μL of MR-(RR)_2_. CTSB activity at the periphery of the cell is indicated with white arrows. (E) Protein levels of CTSB evaluated by western-blot analysis in purified cell surface fraction and washing effluent (20 µL) from TM cells phagocytically challenged for ten days FITC-labeled E. coli. The levels of the non-specific band at 60 kDa served as loading control. Western-blots and confocal images are representative from three different experiments.

Alhough cathepsins are preferentially located within the lysosomal lumen; some specific subtypes, CTSB among them, are known to be expressed on the cell surface as well as secreted into the extracellular space in different cell types, preferentially of mesenchymal origin, either constitutively or under stress conditions [31–35]. Since TM cells are originated from the periocular mesenchyme, we wondered first, whether CTSB could also be expressed on the cell surface and secreted into extracellular media in TM cells; and second, whether phagocytic stress could alter CTSB localization or increase surface expression and/or secretion of the protease. Western-blot analysis indicated that porcine TM cells constitutively secrete the inactive precursor form pro-CTSB. Moreover, the levels of secreted pro-CTSB were markedly elevated upon phagocytosis with E. coli and to a lesser extent with collagen I-coated beads (Figure 6A). Increased CTSB secretion was selective, since phagocytic challenge with E. coli and collagen I-coated beads did not affect the extracellular levels of chitinase 3-like 1, an unrelated protein which is secreted by TM cells [36]. None of the active forms of CTSB could be observed in the culture media. CTSB could also be found in porcine aqueous humor samples, containing both the mature and the processed CTSB (Figure 6B). Cathepsin B activity was not detectable either in culture media or in aqueous humor samples.

Localization of CTSB on the cell surface was investigated in TM cells transiently transfected with a plasmid construct containing CTSB fused to the reporter gene GFP (CTSB-GFP). As seen in Figure 6C, transfected cells showed a punctuate GFP fluorescence signal in the periphery of the cell (white arrows). Note that due to the acidic pH, GFP fluorescence is quenched within the lysosomes (perinuclear region) rendering reduced signal. To investigate whether CTSB present on the cell periphery was active, we did monitor CTSB activity in vivo using the fluorogenic probe cresyl violet linked to a CSTB target sequence peptide (MR-

(RR)_2_). Intense punctuated red fluorescence, resulting from the hydrolysis of the probe, was preferentially observed in the perinuclear region, which corresponds to the subcellular location of lysosomes (Figure 6D). As indicated with the white arrows, CTSB activity was also detectable in the periphery of the cell. It is plausible overexpression of CTSB-GFP to alter normal trafficking or abnormal CTSB localization, therefore we further confirmed CTSB expression on the cell surface by Western-blot analysis in purified cell surface biotinylated fractions. As seen in Figure 6E, TM cells constitutively expressed pro-CTSB on the cell surface, the levels of which significantly increased upon phagocytosis. Although high amounts of scCTSB and dsCTSB were found in the effluent, representing the intracellular fraction, these forms were not detected on the cell surface.

### Intracellular degradation of DQ-Gelatin is located within lysosomes and it is associated with CTSB activity

**Figure 7:**
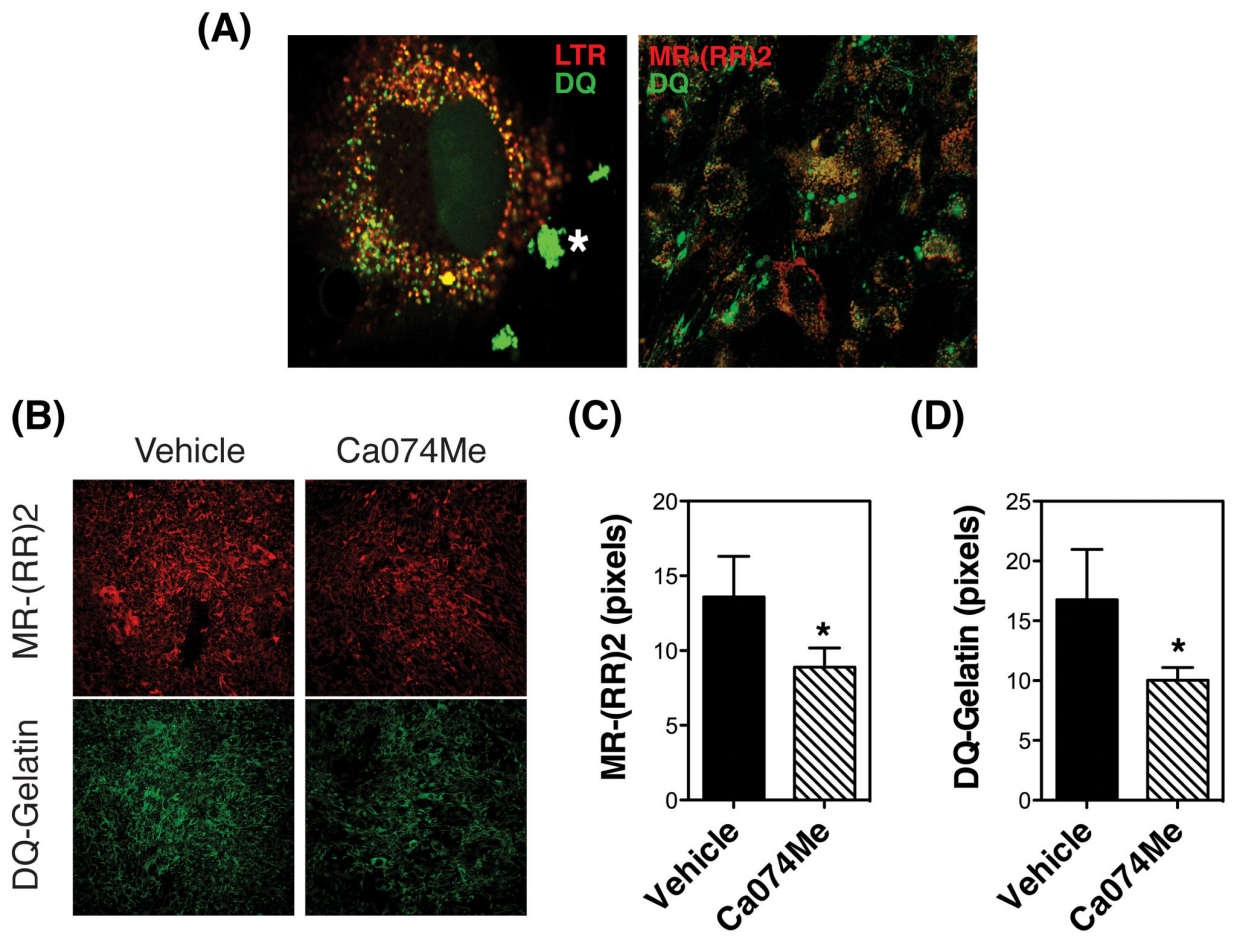
DQ-Gelatin is degraded intracellularly within lysosomes in association with CTSB activity. (A) Porcine TM cells were plated onto Lab-Tek II chambers coated with 20 µg/mL of DQ-gelatin. Two days later, cells were incubated for one hour with LTR (100 nM, red fluorescence, left panel) or MR-(RR)_2_ (red fluorescence, right panel). Green signal indicates fluorescence peptides released by proteolytic degradation of the quenched DQ-gelatin. Co-localization of DQ-degradation products with lysosomes (left panel) or CTSB activity (right panel) is shown as orange/yellow signal. Asterisks (*) indicate the areas where DQ-gelatin is extracellularly degraded. Note that DQ-gelatin degradation products found in the extracellular space do not co-localize with either LTR or MR-(RR)_2_. (B) In vivo CTSB activity in TM cells grown onto onto Lab-Tek II chambers coated with 20 µg/mL DQ-gelatin for one day and exposed for 24 hours to Ca074Me (40 µM). The fluorescent peptides released from the MR-(RR)_2_ proteolytic cleavage (red fluorescence) and from the degradation of DQ-gelatin (green fluorescence) in the presence or absence of Ca074Me were visualized in vivo by confocal microscopy. The fluorescence intensities from five different images were quantified using ImageJ (C, D). Values are mean ± SD. *, p<0.05, ***p<0.001 (t-test, n=5).

It has been historically believed that proteolysis of ECM components occurs extracellularly; however, it is now thought that it primarily occurs at the cell membrane and intracellularly, by endocytosis of partially degraded proteins [20,37–39]. Moreover, several studies have demonstrated the ability of CTSB to contribute to such pericellular and/or intracellular degradation of ECM and basement membrane proteins in some cell types [20,40–44]. To test whether ECM is degraded intracellularly in TM cells, we monitored the proteolytic degradation of DQ-gelatin via a live-cell proteolysis assay. This probe is heavily labeled with FITC molecules so that its fluorescence is quenched. After cleavage by gelatinolytic activity, fluorescent peptides are produced. As shown in Figure 7A (left panel), degradation products of gelatin were observed extracellularly (marked as asteriks), and intracellularly in the perinuclear region, colocalizing with the lysosomal marker LTR. Second, we investigated the participation of CTSB in the proteolytic degradation of gelatin in TM cells by monitoring in vivo the colocalization of DQ degradation products with MR-

(RR)_2_. Intracellular degradation products of DQ-gelatin were observed in vesicles containing active CTSB (Figure 7A, right panel). No co-localization of active CTSB and DQ-gelatin degradation products could be observed extracellularly. Similar results were obtained when using DQ-collagen I and DQ-collagen IV as ECM substrates (Figure S1). To confirm that CTSB activity is indeed required for the degradation of gelatin, in vivo proteolysis was performed in the presence of Ca074Me (40 μM), a selective CTSB pro-inhibitor, which is biologically activated after hydrolysis of the ester by intracellular esterases. As shown in Figure 7B and quantified in Figure 7C, the presence of Ca074Me significantly decreased the levels of active CTSB, as well as the amount of DQ-gelatin degradation products (quantified in Figure 7C). Cells remained attached and no effect in viability was observed with the dosage of inhibitor used, as determined by LDH assay (not shown). These results confirm that gelatin is intracellularly degraded within lysosomes in TM cells, and that such degradation is partially dependent on CTSB activity.

### Phagocytosis Promotes CTSB-mediated ECM Remodeling in TM Cells

**Figure 8:**
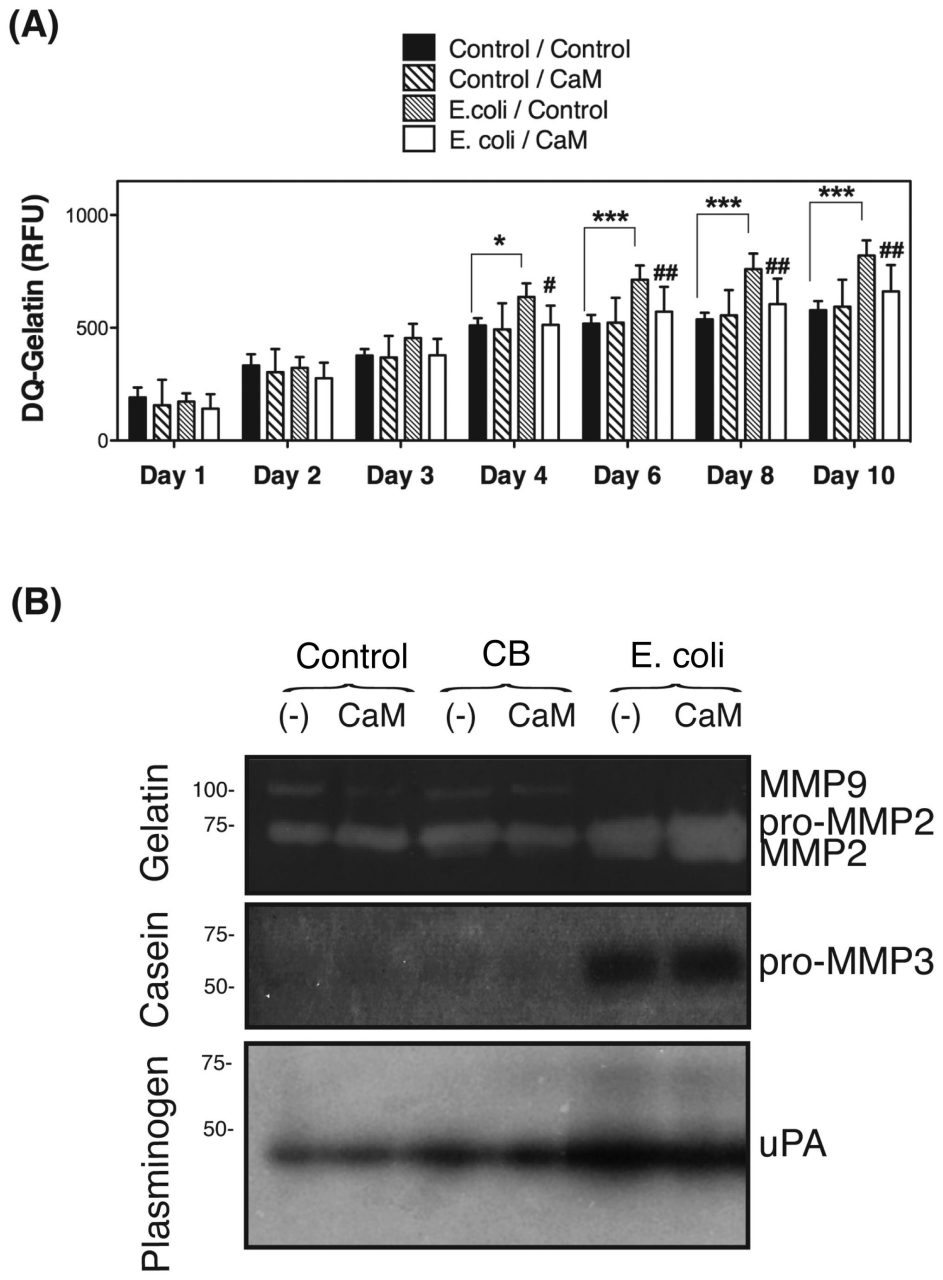
Phagocytosis Promotes CTSB-mediated ECM Remodeling in TM Cells. (A) Confluent cultures of porcine TM cells grown in 96-well plate were phagocytically challenged to E. coli in the presence of vehicle or DQ-gelatin (10 μg/mL), with or without Ca074Me (40 μM). Fluorescence peptides released by the enzymatic cleavage of the substrates were measured in a microplate reader at the indicated times (Em: 495 nm; Exc: 515 nm). All values were corrected for background fluorescence. Values are mean ± SD. * compare E. coli-exposed cultures versus control; ^#^ compare Ca074M-treated cultures versus non-treated, ^*, #^ p<0.05, ^**, ##^ p<0.01, ^***, ###^ p<0.001 (t-test, n=3). (B) Confluent cultures of TM cells were subjected to phagocytic challenge to either E.coli or collagen I-coated beads for ten days. To avoid proteases contained in the serum to interfere with the assays, cells were shifted to serum-free media at day nine after phagocytic challenged. Serum-free cell culture supernatant samples (25 μl) were subjected to gelatin, casein, and plasminogen/casein gel zymography. Areas of proteolytic activity appeared as clear bands. Casein and plasminogen/casein color pictures have been reversed to improve sensititivity using ImageJ.

Our laboratory has recently reported increased collagenolytic and caseinolytic activities in TM cells phagocitically challenged to E.coli [19]. Based on the new data, we wanted to test whether the upregulated expression of CTSB could be responsible for the enhanced collagenolytic and caseinolytic activities upon phagocytosis. For this, TM cells were phagocytically challenged to E. coli in the presence or absence of Ca074Me (40 μM); the degradation products of DQ-gelatin were quantified over time. As shown in Figure 8A, a significant increased in the relative fluorescence associated with DQ-gelatin degradation was observed in phagocytically challenged cultures starting at day four, and continue to rise throughout the length of the experiment (up to nine days). This increase in gelatinolytic activity in TM cells upon phagocytosis was almost entirely prevented with Ca074Me.

We additionally performed substrate gel zymography in conditioned media from TM cells challenged for ten days to E.coli or collagen beads, either in the presence or absence of Ca074Me. To avoid proteases contained in the serum to interfere with the assays, cells were shifted to serum-free media at day nine after phagocytic challenge. Overnight conditioned media was used. Gelatin zymogram showed lytic bands at ~100 kDa, ~73–75 kDa, and at ~62 kDa (Figure 8B). All these bands disappeared when EDTA (MMPs inhibitor) was added to the developer, but experienced no change with PMSF (serine proteases inhibitor) or E64 (cysteine proteases inhibitor), suggesting that the lytic band at ~100 kDa might correspond to MMP9, the one at ~73–75 kDa to pro-MMP2, and the one at ~62 kDa, which was only observed in phagocytically challenge cultures, to MMP2. No qualitative differences could be observed between the cells phagocytically challenged in the presence or absence of Ca074Me. Casein zymography revealed a major band at ~57 kDa in the culture media of TM cells challenged to E.coli, not detected in control cultures. This band disappeared with EDTA but not with either PMSF or E64, which suggests the lytic band to be pro-MMP3. Similarly to MMP2, the expression of MMP3 with E.coli does not seem to be mediated by CTSB. Plasminogen-dependent casein zymography showed a unique lytic band at ~48 kDa that was more intense in phagocytically challenged TM cells, corresponding to urokinase-type plasminogen activator (uPA). The presence of Ca074Me slightly diminished the intensity of uPA in the conditioned media of phagocytically challenged cells. 

## Discussion

In this manuscript we have reported for the first time the specific upregulation and increased secretion of the lysosomal hydrolase CTSB upon phagocytosis with E. coli and collagen I-coated beads. Moreover, we have also demonstrated here that phagocytic challenge promotes increased ECM degradation by mechanisms involving activation of proteases of at least three classes (cysteine proteases, serine proteases, and MMPs). Finally, our data indicate that CTSB is partly responsible for the increase in gelatinolytic activities observed upon phagocytosis in TM cells.

Phagocytosis is central to the degradation of foreign particles. The phagocytic process comprises a variety of events that are initiated by the internalization of the extracellular material into a new compartment generated from the plasma membrane, the phagosome. Newly formed phagosomes do not contain degradative capacity. Through a progressive maturation process that is dependent on the sequential fusion with endosomes and lysosomes, the phagosome acquires acidic pH and lysosomal hydrolytic enzymes, and it is transformed into a phagolysosome, whereby internalized material is ultimately degraded [23–26]. Despite this close relationship between the phagocytic and the lysosomal pathways, very few studies in the literature address whether and how phagocytic challenge might affect the lysosomal cellular function.

In agreement with others, our data demonstrate that TM cells are capable of ingesting a vast variety of materials for an extended period of time without compromising cell viability [5–11]. Although we did not observe a marked preference between phagocytic substrates (opsonized versus non-opsonized, biotic versus nonbiotic), the maturation of phagosomes appears to differ depending on the contained particle. Thus, whereas E.coli-containing phagosomes showed 100% co-localization with the lysosomal marker LTR, thus indicating the maturation into phagolysosomes, inert latex beads did not. We cannot discount that the presence of non-biotic material might affect the uptake of the tracer or the phagolysosomal pH; however, electron micrographs showed that both latex beads and pigment particles preferentially existed within the cells in isolated phagosomes. Interestingly, collagen I-coated beads could be found in different intermediates maturation steps: as isolated phagosomes, non-stained by LTR; as isolated phagolysosomes, displaying LTR fluorescence surrounding the surface membrane; and by electron microscopy, as mature merged autophagolysosomes. Maturation of phagolysosomes seemed to be associated with higher lysosomal content as quantified by LTR and LAMP1 content. Similar results have been reported in macrophages when comparing the maturation of phagosomes containing opsonized sheep erythrocytes, biodegradable poly-e-caprolactone microspheres, and non-biodegradable polystyrene microspheres [24].

Phagocytically challenged TM cells demonstrated an overall increased serine and cysteine cathepsin activities at day two, but decreased afterwards to return to control values in the cultures exposed to non-degradable particles. In contrast, cultures exposed to E. coli. demonstrated sustained elevated protease activity, including serine proteases, several cysteine proteases (CTSB, CTSL, CTSS), and aspartyl proteases. Based on this, it is very tempting to speculate the existence of a cellular mechanism capable of distinguishing and sensing when enhanced degradative capacity is required within phagolysosomes. In this regard, several recent manuscripts have demonstrated a central role of lysosomal efflux permeases, which export breakdown degradation products to the cytosol, in regulating lysosomal function and cellular responses to nutritional stress [45,46]. Similar mechanisms might be used to regulate a cellular response to phagocytosis, depending on whether degradation products are generated or not within phagolysosomes.

Interestingly, qPCR and WB analysis demonstrated higher mRNA and protein levels, respectively, of CTSB in TM cells phagocytically challenged to E. coli. Elevated CTSB was also confirmed, although to a lesser degree, in TM cells exposed to collagen I-coated beads, but not in cultures exposed to carboxylated beads or pigment particles. Similar to other cathepsins, CTSB is synthesized as an inactive precursor (pro-CTSB), which is activated upon arrival to the endosome by proteolytic removal of the propeptide to yield the mature single-chain form (sc-CTSB). Once in the lysosomes, sc-CTSB is further cleaved rendering the double-chain form (dc-CTSB), composed of a heavy-chain and a light chain linked by a disulfide bridge. All the three CTSB forms (pro-CTSB, sc-CTSB, dc-CTSB) were up-regulated and no differences in the ratios among them were observed, indicating proper proteolytic maturation and activation, in agreement with the data obtained using RR-AMC. The finding that CTSB expression was not upregulated upon phagocytosis of inert particles was not entirely surprisingly, since a very recent study reported by our laboratory demonstrated, through comparative gene expression profile and functional network analyses, differential molecular and biological response between TM cells phagocytically challenged to either E.coli or pigment [19]. It is possible that cellular response to foreign particles may vary with the ingestion mechanisms or with the phagocytic receptor. Supporting this, a study has shown that the initial receptor ligand interactions modulate gene expression and phagosomal properties during both early and late stages of phagocytosis [47]. Similarly, studies in insects have also shown that distinct signaling pathways regulate the phagocytic activity of biotic and abiotic components [48,49].

It was not surprising either to find that expression of other cathepsins was not altered with phagocytosis, but activity was, however, elevated (i.e. CTSD); or that, in contrast, mRNA levels were higher, but activity was lower (i.e. CTSL). First, although some of the fluorogenic substrates employed are specifically cleaved by a particular cathepsin (for example, RR-AMC, which is specifically cleaved by CTSB), others (FR-AMC and VVR-AMC) are not and can be hydrolyzed by different proteases. Second, cathepsin activity is the result of several levels of regulation, including transcription, posttranscription processing, translation, glycosylation, trafficking, and binding to cellular endogenous inhibitors [31,50,51]. More intriguing is the fact that phagocytosis seems to selectively up-regulate the expression of CTSB, although we do not rule out that the expression of additional cathepsins not tested in this study can be additionally modulated by phagocytosis.

One important finding is the constitutive cell surface expression and secretion of CTSB by TM cells. Most importantly, the levels of both, membrane-bound and secreted CTSB were significantly elevated in the cultures phagocytically challenged to E. coli and collagen I-coated beads, but not with latex beads or pigment. Moreover, aqueous humor samples revealed the presence of pro-CTSB and mature sc-CTSB. Secretion of CTSB has been described in other cell types either constitutively or induced under certain conditions. In particular, up-regulation and secretion of CTSB is frequently found in several types of malignant cells and cancers [34,35,40,41,43,52,53]. Secretion of CTSB has been also reported to be induced by interactions with matrices [54,55]. How CTSB reaches the cellular surface and the extracellular space is not completely understood. One possibility is CTSB to be re-routed and directed in a retrograde fashion from late endosomes/lysosomes to plasma membrane domains, where it might remain bound to still unidentified membrane receptor or be secreted into the extracellular space [56–58]. However, although some CTSB activity was observed on the cell periphery, most of the enzyme was detected as pro-CTSB, non-processed within the lysosomes on the surface fraction and in the culture media by immunoblots. It is more likely then that TM cells use the same alternative M6P-independent transport route described in macrophages and fibroblasts, and secrete CTSB as zymogen by following the default secretory pathway [59]. Regardless of which alternative route is present in TM cells, our data seem to indicate that phagocytosis does not favor one versus another, but rather increases CTSB expression.

Several studies have shown the ability of CTSB to degrade ECM either intracellularly, extracellularly, or both by initiating a proteolytic cascade that involves uPA, plasminogen/plasmin, and MMPs [20,27,32,40–43,60–62]. Via a live-cell proteolysis assay, we observed that in TM cells degradation products of quenched-fluorescent DQ-gelatin were located intracellularly in the perinuclear region, co-localizing with LTR, in vesicles containing active CTSB. Moreover, intracellular degradation of gelatin was significantly blocked by CA074Me, a cell-permeable intracellular CTSB inhibitor, thus confirming a role of CTSB in the intracellular proteolytic degradation of this substrate. Using a similar approach, we quantified the total (extracellular and intracellular) degradation products of the DQ-gelatin in TM cells challenged to E. coli. Very interesting, our data revealed sustained increased gelatinase activity in phagocytically stressed cultures, which was almost entirely prevented with intracellular inhibition of CTSB. Inhibition of extracellular CTSB by E64 did not have any effect in the proteolytic activity tested (not shown).

Intriguingly, constitutive total degradation levels of DQ-gelatin were not affected by intracellular inhibition of CTSB, suggesting that additional factors induced by phagocytosis are required for CTSB-mediated proteolytic activity. One potential factor might involve activation of CTSB itself. Alternatively, it is possible that ECM components must be first extracellularly pre-digested by other proteases also activated or upregulated by phagocytosis before their up-take for intracellular proteolysis. Supporting this, our laboratory recently reported upregulated expression of MMP1 and MMP3 in phagocytically challenged TM cells [19]. Similarly, in gel zymography of culture media samples showed qualitative differences in the lytic bands corresponding to MMP2, MMP3, and uPA between the control cultures and those phagocytically challenged. Future studies will be aimed at investigating whether these changes are translated into activation of the proteolytic cascade with phagocytosis.

An important aspect to discuss is the physiological significance of our findings. We acknowledge that although E. coli bioparticles are a widely accepted method to trigger phagocytosis, and have been extensively used to study phagocytosis in TM cells, they do not constitute a natural phagocytic ligand for TM cells in vivo, with the exception of some secondary glaucomas (uveitis glaucoma and glaucoma associated with keratitis). Therefore, the fact that the described changes could also be observed upon phagocytosis of collagen I-coated beads are of extremely relevance. Cells in the TM are lining beams of connective tissue made up of various ECM proteins, including collagens. A key role of phagocytosis in collagen turnover and remodeling in connective tissues has been proposed [63]. Whether CTSB could also be upregulated in other phagocytic cells or in TM cells in response to other biotic substrates such as apoptotic cells or cell debris is still to be determined. We should also emphasize here that the relatively inert behavior of pigment particle agrees with our previous data and that reported by others, describing pigment to alter neither trabecular cell function nor morphology [5,8,19]. The mechanisms underlying increased IOP in pigmentary glaucoma are still not understood. While in pigment dispersion syndrome, most of the TM cells phagocytosing pigment granules stay in place, similar to what we have observed in cultured conditions, pigmentary glaucoma is characterized by a loss of TM cells and fusion of the denuded trabecular beams [64]. It is likely the existence of additional mechanisms to trigger the detachment of pigment-overloaded TM cells in vivo conditions.

In summary, we report for the first time here the specific up-regulation and increased levels of membrane-bound and secreted CTSB in TM cells following phagocytic challenge to E. coli and collagen I-coated bead, as well as overall increased CTSB-mediated gelatinolytic activity. This increased proteolytic activity mediated by CTSB, in particular in response to phagocytosis of collagen I, a true phagocytic ligand of TM cells in vivo, might explain the reported detachment of TM cells from the trabecular beams following phagocytosis in vivo and in vitro [5,6,8,15,16], as well as the short-term loss in cell-matrix cohesiveness in cell culture conditions [17,18]. In addition, given the important role of the ECM to the regulation of outflow facility [65–67], our results support a novel role of phagocytosis in the outflow pathway tissue physiology by modulating ECM remodeling.

## Supporting Information

Figure S1 DQ-Collagen I and IV are also degraded intracellularly within lysosomes in association with CTSB activity. Porcine TM cells were plated onto Lab-Tek II chambers coated with 20 µg/mL of DQ-Collagen I or DQ-Collagen IV. Two days later, cells were incubated for one hour with (SM-A) LTR (100 nM, red fluorescence) or (SM-B) MR-(RR)2 (red fluorescence). Green signal indicates fluorescence peptides released by proteolytic degradation of the quenched DQ-products. Co-localization of DQ-degradation products with lysosomes (SM-A) or CTSB activity (SM-B) is shown as orange/yellow signal. Asterisks (*) indicate the areas where DQ-products are extracellularly degraded.
(TIF)
