# Supplementary figures and images for: Cathepsin B Is Up-Regulated and Mediates Extracellular Matrix Degradation in Trabecular Meshwork Cells Following Phagocytic Challenge
Source: PLoS One. 2013 Jul 3;8(7):e68668. doi: 10.1371/journal.pone.0068668 (PMC3700899; doi:10.1371/journal.pone.0068668)

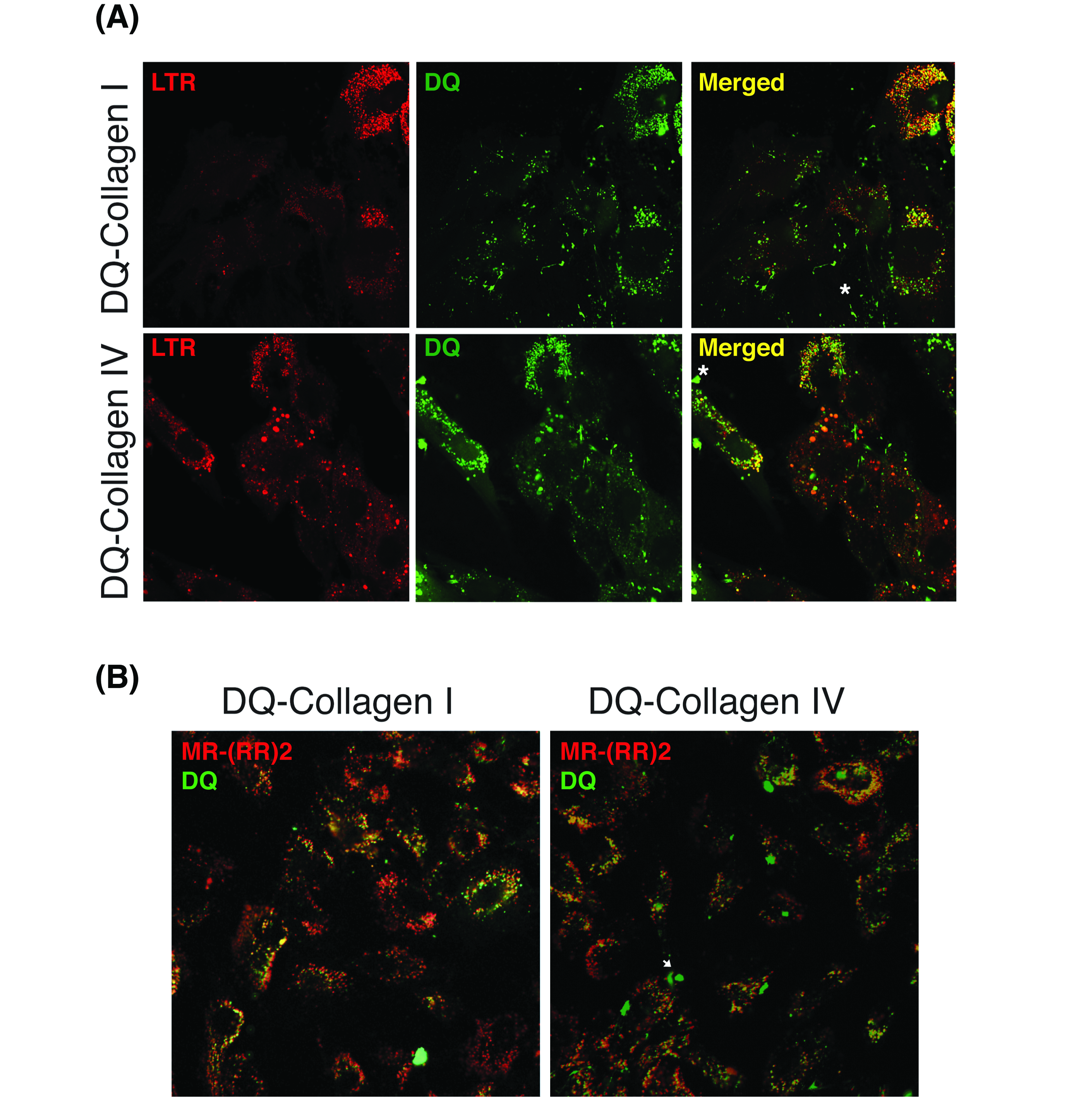

Supplement: Figure S1 — Porcine TM cells were plated onto Lab-Tek II chambers coated with 20 µg/mL of DQ-Collagen I or DQ-Collagen IV. Two days later, cells were incubated for one hour with (SM-A) LTR (100 nM, red fluorescence) or (SM-B) MR-(RR)2 (red fluorescence). Green signal indicates fluorescence peptides released by proteolytic degradation of the quenched DQ-products. Co-localization of DQ-degradation products with lysosomes (SM-A) or CTSB activity (SM-B) is shown as orange/yellow signal. Asterisks (*) indicate the areas where DQ-products are extracellularly degraded. (TIF) [file pone.0068668.s001.tif]
